# PP105 Assessment Of Blood Biomarkers For The Diagnosis Of Alzheimer’s Disease: From Promise To Reality

**DOI:** 10.1017/S0266462324002666

**Published:** 2025-01-07

**Authors:** Carlos Sanchez-Piedra, Mario Cardaba-Arranz, Esther Elena García-Carpintero, Inaki Imaz-Iglesia

## Abstract

**Introduction:**

The objective of this assessment was to assess diagnostic effectiveness of phosphorylated isoforms of the tau protein (p-tau) in blood samples (plasma and/or serum) in Alzheimer’s disease diagnosis compared to clinical criteria, cerebrospinal fluid (CSF) biomarkers, positron emission tomography (PET), or post-mortem pathology imaging.

**Methods:**

A systematic review of MEDLINE, Cochrane, and Embase was undertaken from 1 January 2000 to 1 September 2022. QUADAS-2 was used to assess the quality of the studies. The selection and quality assessment was carried out independently by two reviewers.

**Results:**

A total of 1,811 references were identified after eliminating duplicates, selecting five: two studies evaluated p-tau181, two others evaluated p-tau217, and one evaluated both isoforms. The studies provided information on diagnostic accuracy using ROC curves. However, none of the studies provided information on sensitivity and/or specificity. The ROC curve values ranged from 83.3 percent to 99.4 percent for p-tau181 and 84 percent to 94 percent for p-tau217. Information was presented in an unclear and heterogeneous way, so our PICO (population, intervention, comparator, outcome) questions were left unanswered. The heterogeneity was due to factors such as the type of isoform, the laboratory platform, and the classification criteria considered.

**Conclusions:**

Despite technology presenting as promising in several studies, the available evidence did not provide high-quality information about its diagnostic accuracy. Diagnostic technologies are often launched and marketed without enough high-quality evidence. The generation of evidence in diagnostic studies is often based on designs that do not provide adequate health technology assessment information.

